# TALEN-Interceded Genome Editing in Plants: Unveiling New Frontiers in Secondary Metabolite Improvement and Genetic Diversity

**DOI:** 10.3390/plants14193024

**Published:** 2025-09-30

**Authors:** Wajid Zaman, Atif Ali Khan Khalil, Adnan Amin

**Affiliations:** 1 Department of Life Sciences, Yeungnam University, Gyeongsan 38541, Republic of Korea; wajidzaman@yu.ac.kr; 2 Department of Biotechnology, Yeungnam University, Gyeongsan 38541, Republic of Korea; atif.ali@yu.ac.kr

**Keywords:** TALENs, alkaloids, FokI nuclease, genome editing, multi-omics approaches

## Abstract

Secondary metabolites, including alkaloids, flavonoids, and tannins, are crucial for human health, agriculture, and ecosystem functioning. Their synthesis is often species-specific, influenced by both genetic and environmental factors. The increasing demand for these compounds across various industries highlights the need for advancements in plant breeding and biotechnological approaches. Transcription activator-like effector nucleases (TALENs) have emerged as a powerful tool for precise genome editing, offering significant potential for enhancing the synthesis of secondary metabolites in plants. However, while plant genome editing technologies have advanced significantly, the application of TALENs in improving secondary metabolite production and expanding genetic diversity remains underexplored. Therefore, this review aims to provide a comprehensive analysis of TALEN-mediated genome editing in plants, focusing on their role in enhancing secondary metabolite biosynthetic pathways and improving genetic diversity. The mechanisms underlying TALENs are examined, including their ability to target specific genes involved in the synthesis of bioactive compounds, highlighting comparisons with other genome editing tools such as CRISPR/Cas9. This review further highlights key applications in medicinal plants, particularly the modification of pathways responsible for alkaloids, flavonoids, terpenoids, and phenolic compounds. Furthermore, the role of TALENs in inducing genetic variation, improving stress tolerance, and facilitating hybridization in plant breeding programs is highlighted. Recent advances, challenges, and limitations associated with using TALENs for enhancing secondary metabolite production are critically evaluated. In this review, gaps in current research are identified, particularly regarding the integration of TALENs with multi-omics technologies and synthetic biology approaches. The findings suggest that while underutilized, TALENs offer sustainable strategies for producing high-value secondary metabolites in medicinal plants. Future research should focus on optimizing TALEN systems for commercial applications and integrating them with advanced biotechnological platforms to enhance the yield and resilience of medicinal plants.

## 1. Introduction

Genetic diversity is essential for plant improvement, serving as the foundational resource for developing traits that support adaptation to changing environmental conditions, resistance to pests, and improved yield [1,2]. Greater genetic variation within a crop species increases the risk of identifying traits that confer tolerance to both biotic and abiotic stresses [3]. However, traditional breeding methods, relying on crossing genetically diverse parents, are often time-consuming and constrained by the limited genetic diversity available in parental lines [4]. In contrast, genome editing in plants offers a promising solution to these limitations by enabling precise and targeted modifications to plant genomes. This technology has transformed agricultural biotechnology and is crucial for addressing complex challenges such as improving crop yield, stress tolerance, and nutritional value. While traditional breeding techniques remain valuable, they are limited by the slow pace of genetic improvement and the lack of precision in selecting desirable traits [5]. The advent of genome editing technologies, such as zinc finger nucleases (ZFNs), transcription activator-like effector nucleases (TALENs), and CRISPR-Cas9, has enabled researchers to introduce targeted modifications at specific genomic loci, thereby accelerating the breeding process [6]. Among these tools, TALENs have gained prominence for their high specificity and efficiency in plant systems [7].

TALENs comprise a DNA-binding domain derived from transcription activator-like effectors (TALEs), which are proteins naturally produced by certain plant-pathogenic bacteria [8]. These effectors can be engineered to recognize and bind specific DNA sequences, while the FokI nuclease domain introduces a double-strand break at the target site, facilitating precise genetic modification [9]. In contrast to other genome editing methods, TALENs enable precise genetic modifications with minimal off-target effects, making them a powerful tool for crop improvement [10]. TALEN-based genome editing offers an accurate and efficient approach to introducing novel genetic variations into plant populations without the need for extensive crossbreeding [11]. TALENs introduce specific mutations or regulatory changes leading to novel traits such as improved disease resistance, drought tolerance, and nutrient content [12]. This targeted mutagenesis strategy represents a significant advancement over traditional breeding, as it generates genetic diversity at specific loci rather than relying on the randomness of traditional crossbreeding methods.

Beyond their agricultural applications, TALENs hold significant value in medicinal plant research and commerce, particularly for enhancing the production of plant secondary metabolites [13,14]. The significance of secondary metabolites in plants is well recognized, as these compounds are crucial for plant defense, growth regulation, and reproduction [15]. Secondary metabolites, such as alkaloids, terpenoids, flavonoids, and phenolic compounds, are synthesized through complex biosynthetic pathways involving multiple enzymes and genetic interactions [16]. These compounds are essential for plant physiology and their pharmacological and industrial relevance to humans. For example, alkaloids are widely utilized in medicine for their analgesic and anticancer properties [17], while flavonoids are recognized for their antioxidant, anti-inflammatory, and anticancer activities [18]. However, the biosynthesis of these valuable metabolites is often limited by the genetic potential of the producing plant species [19]. Genome editing, particularly using TALENs, now enables precise manipulation of key genes within biosynthetic pathways, thereby enhancing the production of secondary metabolites [20]. Targeting specific genes involved in the regulation or biosynthesis of secondary metabolites using TALENs can optimize plant varieties for higher yields of these bioactive compounds, thereby contributing to advancements in the plant-based pharmaceutical and nutraceutical industries [21]. While earlier reviews have explained the extensive applications and potential of TALENs in plant genome editing, this review provides a more focused analysis of their role in enhancing secondary metabolites synthesis and promoting genetic diversity of medicinally important species. It precisely scrutinizes the potential of TALENs’ to amend complex biosynthetic pathways for enhanced bioactive compounds production such as alkaloids, flavonoids, and terpenoids, which are crucial for medicinal and agricultural applications. Moreover, the review highlights recent advancements in TALEN technology, particularly its integration with multi-omics and synthetic biology approaches, offering novel opportunities to enhance the yield and resilience of medicinal plants. In comparing TALENs with other genome editing tools like ZFNs, CRISPR/Cas9, we identify the unique advantages of TALENs in terms of specificity and precision. This review also addresses existing research gaps and proposes strategies to overcome challenges in TALEN-mediated metabolic engineering. This review aims to provide a detailed understanding of how TALENs contribute to the development of more resilient and high-yielding plant varieties through an examination of their applications, with broad implications for agriculture, medicine, and industry.

## 2. Mechanisms and Recent Advancements in Transcription Activator-like Effector Nuclease Technology

TALEN technology has undergone continuous improvements over the years, particularly with respect to efficiency, specificity, and ease of use [22]. These advancements have expanded the potential for complex genetic modifications, including the modification of entire metabolic pathways and the simultaneous enhancement of multiple agronomic traits within a single plant variety.

### 2.1. Structural Composition and Mechanism of Transcription Activator-like Effector Nuclease

TALENs comprise two primary components: a TALE-derived DNA-binding domain and a FokI nuclease domain [12]. The DNA-binding domain consists of several tandemly repeated motifs, each recognizing a single base pair within the target DNA sequence [23]. These repeats vary in their amino acid composition, particularly at positions 12 and 13, determining the binding specificity of TALE–DNA interaction. The modularity of these repeat regions enables precise targeting of virtually any DNA sequence [8,24].

### 2.2. Transcription Activator-like Effector Nuclease-Mediated Gene Editing Process

**Figure 1:**
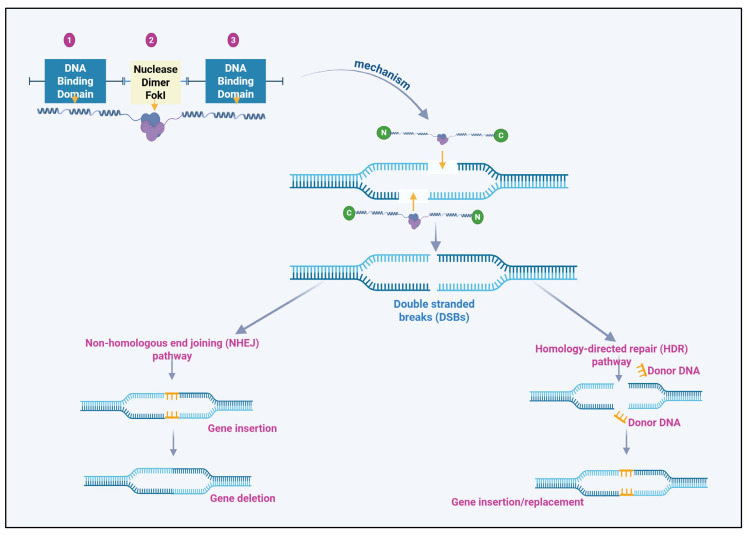
Structure and mechanism of TALENs (Transcription Activator-Like Effector Nucleases) in genetic engineering. This figure illustrates the structure and mechanism of TALENs used in genetic engineering.

Once TALENs bind to their target DNA sequence, the FokI nuclease domain, a type II restriction enzyme within the TALEN construct, dimerizes to induce a double-strand break at the specified site [25]. This break activates the endogenous DNA repair pathways of the plant, proceeding through non-homologous end joining (NHEJ) or homology-directed repair (HDR) [26]. NHEJ is an error-prone repair pathway often resulting in insertions or deletions (indels) at the break site, thereby generating gene knockouts [27]. In contrast, HDR enables precise genetic modifications when a donor template is available. This ability to induce specific genetic changes at precise loci enables TALENs to be used for enhancing or silencing genes involved in secondary metabolite pathways [28]. TALENs significantly increase the production of secondary metabolites by disrupting or enhancing the function of specific genes (Figure 1).

A defining feature differentiating TALENs from other genome-editing tools such as ZFNs and CRISPR-Cas9 is the customizable nature of the TALE DNA-binding domain [29]. In ZFNs, the DNA-binding domain comprises zinc finger motifs, offering less flexibility in sequence recognition than TALE repeat arrays [30]. Each zinc finger recognizes a specific sequence of three base pairs; however, designing zinc fingers to target unique DNA sequences is often challenging [30]. In contrast, TALE repeats offer greater flexibility, as each repeat binds to a single base pair [31]. This modularity makes TALENs more versatile and easier to design for a broader range of genomic targets. Additionally, the requirement for FokI nuclease domain dimerization ensures that nuclease activity remains highly localized to the target site [32], thereby enhancing the overall precision of TALEN-mediated genome editing.

The efficiency of TALENs in inducing targeted genetic modifications is influenced by the activity of the FokI nuclease domain [12]. Consequently, the specificity of TALENs is attributed to the DNA-binding domain and the precise positioning of the double-strand break [33]. Introducing a double-strand break at the target DNA site is a critical step for initiating the cellular repair processes leading to gene editing. Recent advances in TALEN design have focused on enhancing the efficiency of DNA cleavage by optimizing the FokI nuclease domain and its dimerization dynamics [34]. Modifications to the FokI domain, including alterations in its cleavage activity, have enabled the enhancement of TALENs for inducing targeted mutations in plant genomes (Figure 1).

### 2.3. Comparisons of Transcription Activator-like Effector Nucleases with Other Genome Editing Tools

TALENs offer several advantages over other genome editing technologies, particularly CRISPR-Cas9 and ZFNs. A key advantage is their high specificity. While CRISPR-Cas9 is a widely used genome editing system, it relies on short guide RNA (gRNA) sequences to direct the Cas9 nuclease to the target DNA. This RNA-based targeting occasionally results in off-target effects when the gRNA partially matches unintended genomic regions [35]. In contrast, TALENs rely on protein-DNA interactions, which are generally more specific due to their DNA-binding domains being engineered to recognize highly precise target sequences [36,37]. This high level of specificity is particularly beneficial when editing genes in complex plant genomes with off-target mutations potentially leading to undesirable traits or confounding experimental outcomes. Furthermore, TALENs are less likely to induce genomic rearrangements or insertions at off-target sites, making them a valuable tool for precise genome editing in plants [38].

Another significant advantage of TALENs over ZFNs and CRISPR-Cas9 is their ability to target a broader range of DNA sequences [6,39]. For example, CRISPR-Cas9 is limited by the requirement for protospacer adjacent motifs (PAMs) near the target site, restricting its applicability in certain genomic regions. In contrast, TALENs are not dependent on specific sequence motifs [40]. The modular design of TALE repeat domains enables TALENs to recognize and bind almost any DNA sequence, providing greater flexibility in genome targeting regardless of PAM availability [23]. This flexibility is particularly valuable when working with plant species that exhibit complex or poorly characterized genomes, with PAM sequences potentially absent in regions of interest [41]. Additionally, TALENs are effective in various plant species, including both dicots and monocots [41]. In contrast, the performance of CRISPR-Cas9 is more variable among plant species, primarily due to its dependence on specific PAM sequences [42].

Despite these advantages, TALENs present several challenges. Their construction involves synthesizing large, custom-designed DNA-binding domains, a process both time-consuming and cost-intensive [12]. Furthermore, optimizing TALENs for different target sites often requires iterative design and validation [43]. While TALENs offer high specificity, their construction and delivery remain more labor-intensive than that of CRISPR-Cas9, gaining more adoption due to their simpler and user-friendly design [44]. However, for applications with critical precision and minimized off-target effects, TALENs remain a valuable tool in plant genome editing (Table 1).

## 3. TALENs and Secondary Metabolite Synthesis

Secondary metabolites are plant-derived organic compounds that, while not directly involved in growth, development, or reproduction [50], play crucial roles in environmental interactions, such as defense against pathogens, herbivores, and stress [51]. These compounds also facilitate plant communication and have evolved to enhance the survival and ecological adaptability of plants [52]. Secondary metabolites are classified into alkaloids, terpenoids, flavonoids, and phenolic compounds based on their chemical structures [53]. Each class is synthesized through distinct biosynthetic pathways, many of which are tightly regulated by complex gene networks [54]. However, the yield of these secondary metabolites is often limited by plant genetics, and traditional breeding methods rarely enhance their production [55]. Given their importance, enhancing secondary biosynthesis through genetic modifications is a key goal in plant biotechnology. TALEN technology offers a precise gene editing tool for targeting specific gene pathways, thus enhancing secondary metabolite production in plants.

### 3.1. Biosynthesis of Secondary Metabolites

#### 3.1.1. Terpenoid Pathways

Terpenoids, also known as isoprenoids, constitute one of the largest and most diverse classes of plant secondary metabolites, functioning in defense, attraction, and protection [56]. They originate from two major biosynthetic pathways: the mevalonate (MVA) and methylerythritol phosphate (MEP) pathways [57]. The MVA pathway occurs in the cytosol, while the MEP pathway is localized in plastids [58]. Both pathways produce isoprenoid precursors—isopentenyl diphosphate and dimethylallyl diphosphate—which serve as terpenoid building blocks [59]. These precursors undergo enzymatic conversion into diverse terpenoids, including monoterpenes, sesquiterpenes, diterpenes, and triterpenes, many possessing medicinal, aromatic, or insecticidal properties [60]. The regulation of these pathways involves transcription factors, enzymes, and metabolites that modulate key gene expression. TALENs can be used to target specific genes within these pathways, such as those encoding enzymes, including geranyl diphosphate synthase (GPPS) or terpene synthases, thereby enhancing desired terpenoid production [61].

#### 3.1.2. Alkaloid Biosynthesis

Alkaloids are nitrogen-containing secondary metabolites with diverse biological activities, including antimicrobial, analgesic, and psychoactive effects [62]. Alkaloids are primarily biosynthesized from amino acids such as tyrosine, tryptophan, and phenylalanine, which serve as precursors [63]. Enzymes such as tyrosine decarboxylase, tryptophan decarboxylase, and others catalyze the conversion of these amino acids into intermediate compounds, which are further modified into final alkaloids [64]. Alkaloid production is regulated by a network of transcription factors—MYB, bHLH, and NAC family proteins—that coordinate gene expression in the biosynthetic pathway [65]. TALENs can be employed to manipulate these transcription factors or specific biosynthetic enzymes, such as those in the shikimate or phenylpropanoid pathways, to enhance alkaloid production [66].

#### 3.1.3. Flavonoid and Phenolic Acid Pathways

Flavonoids and phenolic acids are key plant secondary metabolites with antioxidant properties that protect against oxidative stress, UV radiation, and pathogens [67]. Flavonoid biosynthesis begins with the amino acid phenylalanine, which is converted into cinnamic acid and then further modified into precursors such as chalcones [68]. These intermediates are then converted into various flavonoid compounds such as anthocyanins, flavonols, flavones, and isoflavonoids through the activity of enzymes including chalcone synthase (CHS) and flavonoid 3′-hydroxylase (F3′H) [69]. Similarly, phenolic acids such as cinnamic, ferulic, and p-coumaric acids originate from the shikimate pathway and play key roles in plant defense and stress responses [70]. TALENs can be used to target the expression of key enzymes in these pathways, such as CHS, F3′H, and other related genes [71].

### 3.2. Targeting Key Genes in Secondary Metabolite Pathways

#### 3.2.1. Engineering Transcription Factors

Transcription factors critically regulate the expression of genes involved in secondary metabolite biosynthesis [72]. These proteins bind to specific DNA promoter sequences to activate or repress target gene expression [73]. Modulating transcription factor activity is key to regulating metabolite flow through secondary metabolic pathways. TALENs can be used to knock out or activate genes encoding these transcription factors, thus influencing metabolite biosynthesis [74]. For instance, in *Arabidopsis thaliana*, TALENs can be employed to modify the expression of transcription factors such as MYB and bHLH, which regulate flavonoid production [75]. Enhancing the expression of these factors can significantly increase flavonoid production, such as anthocyanins. Similarly, in other plant species, TALENs can be used to fine-tune the regulation of enzymes involved in terpenoid and alkaloid biosynthesis by editing genes encoding their corresponding transcription factors [76]. This approach enables precise control of metabolic networks to produce specific secondary metabolites in desired quantities.

#### 3.2.2. Modulating Enzyme Activity

**Figure 2:**
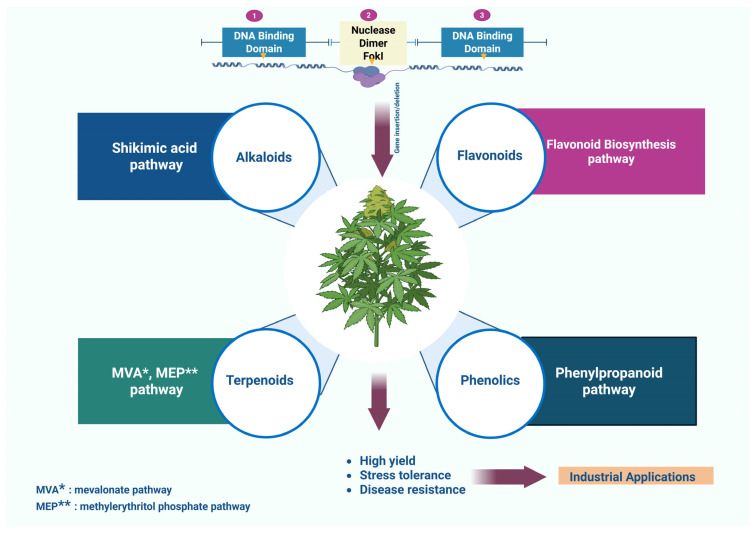
Effect of TALENs (Transcription Activator-Like Effector Nucleases) on enhanced Secondary metabolites production using Shikimic acid, flavonoid biosynthesis, MVA (Mevalonate), MEP (Methylerythritol Phosphate) and phenylpropanoid pathways.

In addition to targeting transcription factors, TALENs can also be used to modify genes encoding enzymes directly involved in secondary metabolite biosynthesis [77]. Enzymes such as CHS, Leucoanthocyanidin Reductase, Chalcone Isomerase in the flavonoid pathway, along with GPPS, Farnesyl Diphosphate Synthase, and Squalene Synthase in the terpenoid pathway, catalyze key reactions controlling carbon flow through these metabolic networks [78,79]. Similarly, various enzymes, including Tyrosine Decarboxylase, N-methyltransferases, Dihydropyrrolizidine Synthase involved in Alkaloids biosynthesis [80] are key TALEN targets. Studies on microorganisms report successful secondary metabolite overproduction using TALENs modulating enzyme activity. For instance, in an investigation, heterodimeric TALENs were designed to simultaneously edit the *FAA1* and *FAA4* genes encoding acyl-CoA synthetases in *S. cerevisiae*. Functional yeast double knockouts generated using these TALENs overproduce large amounts of free fatty acids intracellularly [81]. This precise control over enzyme activity using TALENs enables fine-tuning of secondary metabolite production, offering a powerful method to boost bioactive compound yields (Figure 2; Table 2).

### 3.3. Case Studies of Transcription Activator-like Effector Nucleases in Secondary Metabolite Synthesis

Several successful case studies report the potential of TALENs for enhancing plant secondary metabolite production. In one significant example, TALENs were used to enhance the *Arachis hypogaea* (peanuts) oil quality by targeting the *FAD2* gene, which converts oleic acid to linoleic acid. The oleic acid content in peanut oil is crucial for its health benefits and shelf-life, as higher levels improve stability and reduce oxidation. The study reports the first successful use of TALENs to edit *FAD2*, significantly increasing oleic acid levels and reducing linoleic acid content [86].

Another case study employed CRISPR/Cas9 and TALENs to create heritable mutations in small RNA processing genes in *Glycine max* (soybean) and *Medicago truncatula*. Targeted genes included *GmDrb2, GmDcl3a,* and *GmHen1* in soybean and *MtHen1* in *Medicago truncatula*. Researchers used CRISPR/Cas9 to successfully generate bi-allelic mutations in *GmDrb2a and GmDrb2b* genes, which were heritably transmitted to progeny. TALENs were used to mutate *GmDcl2b*, demonstrating the effectiveness of both platforms. However, some mutations, such as in *GmDcl3a*, were not transmitted to the next generation, highlighting challenges in mutagenesis efficiency. This study enhances the toolkit for studying small RNA biology and provides valuable resources for future functional genomics in legumes [87].

Similarly, TALENs were applied to edit the caffeic acid *O-methyltransferase (COMT)* gene in *Saccharum officinarum*, a crucial gene in lignin biosynthesis. Targeted mutations in COMT reduced lignin content by up to 32% in some mutant lines compared to wild-type plants. This reduction was positively correlated with higher COMT mutation frequencies and improved cell wall characteristics, including elevated hemicellulose and reduced S-lignin subunits, enhancing biomass suitability for bioethanol production. These TALEN-induced mutations were consistently transmitted to vegetative progeny [88].

TALEN-mediated genome editing in *Glycine max* (soya bean) introduced mutations in two fatty acid desaturase genes (FAD2-1A and FAD2-1B), reducing oleic acid conversion to linoleic acid. Consequently, soybean lines with mutations in both genes showed a significant increase in oleic acid (20–80%) and a decrease in linoleic acid (from 50% to under 4%). Some plants lacked the TALEN transgene but carried only the desired mutations. The findings indicate that TALENs can rapidly create valuable crop improvement traits by precisely modifying gene families within a single generation [89].

Glycoproteins (medicinally important) produced in plants contain N-glycans with plant-specific residues—core α(1,3)-fucose and β(1,2)-xylose—that significantly affect their activity, stability, and immunogenicity. TALENs were used to knock out specific genes in *Nicotiana benthamiana* to produce glycoproteins with modified N-glycans. These plants, lacking core α(1,3)-fucose and β(1,2)-xylose residues, showed improved capacity to produce biopharmaceuticals with altered glycosylation patterns. TALENs were used to target two α(1,3)-fucosyltransferase (FucT) and two β(1,2)-xylosyltransferase (XylT) genes, inducing mutations in a significant proportion of regenerated plants. The knockout plants exhibited N-glycans with reduced core α(1,3)-fucose levels and no β(1,2)-xylose. The study also showed that the recombinant rituximab from these mutant plants had a 55% increase in the desired glycoform lacking core α(1,3)-fucose and β(1,2)-xylose. Thus, TALENs enable multiplexed gene editing to produce high-quality biopharmaceuticals [90].

*Oryza sativa* or fragrant rice is prized for its pleasant aroma, primarily caused by 2-acetyl-1-pyrroline (2AP), synthesized due to a defective badh2 allele encoding betaine aldehyde dehydrogenase (BADH2). In a study [91], TALENs were used to target and disrupt the OsBADH2 gene in rice, producing six heterozygous mutants from 20 transgenic lines with these mutations heritable in subsequent generations. The T1 mutant rice lines showed increased 2AP content (0.35–0.75 mg/kg), similar to a positive control variety with the badh2-E7 mutation. Additionally, multiple TALEN pairs targeting different rice genes were introduced, producing mutations in one, two, or all three genes. These findings indicate that TALEN-based targeted mutagenesis is a powerful tool for creating desirable agronomic traits in crops.

## 4. TALENs and Genetic Diversity

Genetic diversity is crucial for improving crops by enabling the breeding of plants that better adapt to environmental stresses, resist diseases, and yield higher-quality produce [92]. As explained earlier, a major advantage of using TALENs for genetic improvement is their precise ability to induce specific mutations [12]. Additionally, TALENs can be used to create genetic diversity in crop populations by introducing specific mutations in key regulatory genes, producing new variations absent in the parent population [93]. This controlled, efficient generation of genetic diversity marks a significant advancement over traditional breeding approaches.

### 4.1. Enhancing Genetic Diversity with Transcription Activator-like Effector Nucleases

Enhancing genetic diversity using TALENs is a key application of this technology in crop improvement. Unlike traditional breeding methods, which rely on natural variation in a population [94], TALENs allow targeted mutations in genes regulating key agronomic traits [95]. This precise approach allows breeders to control genetic diversity, particularly in crops with extensive inbreeding or limited genetic variation. By introducing specific mutations in genes for disease resistance, stress tolerance, and yield, TALENs expand the genetic pool for future breeding [96]. This approach is especially beneficial for crops limited by past breeding practices, by introducing new genetic material without extensive crossbreeding or undesirable traits [97]. For instance, targeting a single stress-tolerance gene with [98] TALENs can generate drought-resistant variants without compromising traits [99], such as yield or disease resistance [96]. Moreover, multiplex TALENs enable simultaneous edits, allowing the development of plants with combined beneficial traits, such as enhanced resistance to pests and improved nutrient content, thereby broadening crop genetic diversity [100].

Similarly, the “Time Factor” is a crucial factor in crop breeding—especially in crops with large or polyploid genomes, such as wheat or barley—where traditional breeding can be time-consuming and inefficient due to the complexity of selecting traits across multiple genome copies [101]. However, TALENs enable precise gene editing within these genomes, accelerating the development of improved varieties [102]. In addition, TALENs can be used to introduce genetic diversity into previously uncharacterized or underexplored crops, broadening the genetic resources available for breeding.

### 4.2. Case Studies in Crop Improvement

TALENs have been successfully applied to improve key agronomic traits and genetic diversity in several major crops. In a prominent case, TALENs were used to modify the *rice* genome to improve its resistance to bacterial blight, a major global threat to rice production. By targeting a specific gene linked to the immune response of plants, researchers developed rice plants with enhanced resistance to the bacterium *Xanthomonas oryzae* without affecting other important traits such as growth and yield. This example highlights the power of TALENs to enhance disease resistance in plants, a critical factor in maintaining food security amid increasing pest and pathogen pressures [103].

Another example of TALEN-driven crop improvement is the modification of *maize* for enhanced drought tolerance. Drought poses a significant challenge to global agriculture, making drought-tolerant crops crucial for maintaining food production in water-scarce regions [104]. TALENs have been used to modify the expression of genes regulating water retention and osmotic balance, producing maize plants that are better able to withstand water stress [105,106]. Precise editing of maize genes controlling water response produced plants with improved drought tolerance without yield loss.

Integrated TALEN and CRISPR-associated (Cas) systems have become powerful genome-editing tools across species. This study reported for the first time targeted mutagenesis in *Zea mays* using TALENs and the CRISPR/Cas system. We designed five TALENs targeting four genes, namely *ZmPDS*, *ZmIPK1A*, *ZmIPK*, and *ZmMRP4*, achieving up to 23.1% targeting efficiency in protoplasts, and approximately 13.3–39.1% somatic mutations in transgenic plants. Furthermore, two gRNAs targeting the *ZmIPK* gene in maize protoplasts were constructed at frequencies of 16.4% and 19.1%, respectively. In addition, the CRISPR/Cas system induced targeted mutations in *Z. mays* protoplasts with efficiencies (13.1%) similar to those obtained with TALENs (9.1%), indicating that both tools enable genome modification in maize [107].

TALENs have also been employed to improve wheat disease resistance and grain quality [108]. Wheat, a staple crop, is vulnerable to diseases, including wheat rust and Fusarium head blight. Using TALENs to target genes involved in the mechanisms of plants, researchers created wheat varieties with enhanced disease resistance [109]. Additionally, TALENs and CRISPR/Cas integrated systems have been used to enhance the nutritional quality of wheat by targeting genes involved in protein and micronutrient synthesis [110]. The ability to modify multiple genes simultaneously has enabled the development of wheat varieties with disease resistance and improved nutritional profiles, such as higher levels of essential amino acids and vitamins. These case studies demonstrate the versatility of TALENs in improving crops, from major staples such as rice and wheat to more specialized species, further highlighting their potential to enhance genetic diversity and crop performance. This also paves the way for developing genetically diverse medicinal plants varieties [111].

## 5. TALENs and Plant Stress Tolerance Mechanisms

The ability to withstand environmental stresses is crucial for plant survival, productivity, and adaptability [112]. Stress tolerance mechanisms enable crops to maintain optimal growth and yield under suboptimal conditions, such as drought, salinity, extreme temperatures, and pathogen attack [113]. Environmental stresses often decrease photosynthesis, nutrient uptake, and damage cellular structures, ultimately lowering crop productivity [114]. Traditionally, stress tolerance improvement in plants relied on selective breeding, which has limitations, particularly in enhancing multiple stress tolerance traits simultaneously. Here, TALENs offer the advantage of introducing specific mutations in target genes, improving plant resilience without extensive crossbreeding.

### 5.1. Introduction to Plant Stress Tolerance

Plant stress tolerance is defined as the ability of plants to maintain normal physiological functions under suboptimal environmental conditions [112]. Stressors can be classified into two categories: abiotic and biotic stress. Abiotic stress includes environmental factors such as drought, salinity, extreme temperatures, and soil acidity that directly affect plant growth, development, and productivity [115]. For example, drought stress reduces water availability, causing plant cell dehydration, osmotic imbalance, and impaired nutrient uptake [116]. Similarly, salinity stress causes toxic sodium ion buildup in plant tissues, disrupting cellular processes [117]. In contrast, biotic stress arises from the presence of pathogens, including bacteria, viruses, fungi, and nematodes, that invade plant tissues and disrupt growth and development [118]. To overcome these stresses, plants have developed sophisticated mechanisms, including stress-responsive genes, signaling pathways, and physiological adaptations, such as osmoprotectant accumulation, antioxidant activation, and ion transport changes [119,120]. Understanding these mechanisms is essential for enhancing crop stress tolerance and ensuring sustainable agriculture.

Abiotic stress tolerance is typically governed by complex gene networks that interact to regulate plant responses [121]. For instance, during drought conditions, plants typically synthesize phytohormones such as abscisic acid, which help in regulating stomatal closure to minimize water loss through transpiration [122]. Additionally, stress-responsive genes, such as those encoding heat shock proteins (HSPs), are upregulated to protect cellular structures from damage under heat stress conditions [123]. Similarly, in response to high salinity, plants may activate pathways that increase osmotic regulator production, such as proline, which helps cells retain water and maintain turgor pressure [124]. These mechanisms involve complex signaling networks that connect environmental stimuli to specific stress responses at the molecular, cellular, and physiological levels. The ability to manipulate these pathways using genome editing techniques such as TALENs offers a powerful opportunity to enhance stress tolerance in crops, enabling them to perform optimally under adverse environmental conditions.

**Figure 3:**
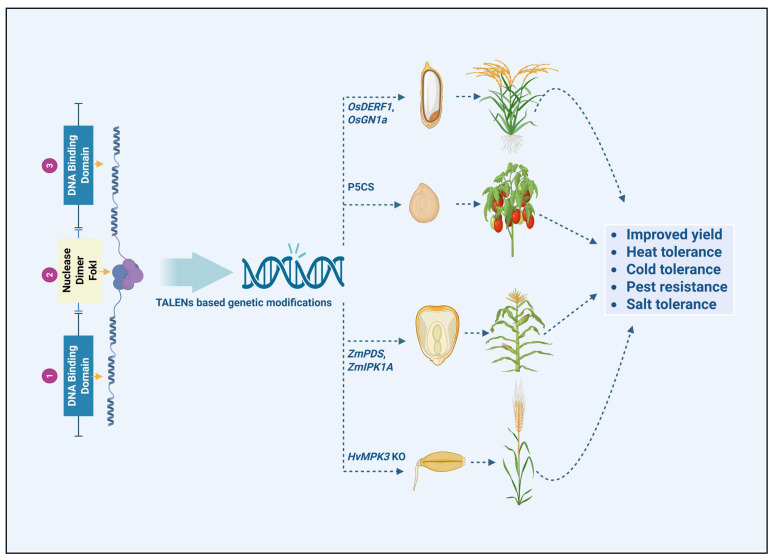
Role of TALENs (Transcription Activator-Like Effector Nucleases) in enhancing genetic diversity and stress tolerance in agricultural crops.

Biotic stress tolerance in plants is governed by a complex defense system that detects and responds to pathogen invasion [125]. Upon infection, plants initiate a series of immune responses, including recognizing pathogen-associated molecular patterns by pattern recognition receptors, which in turn activate downstream signaling pathways [126]. These signaling pathways activate the expression of defense-related genes, such as those encoding antimicrobial peptides, secondary metabolites, and enzymes involved in cell wall reinforcement [127]. Activating these responses can restrict pathogen spread and minimize tissue damage. However, many pathogens have evolved strategies to evade or suppress plant immune responses, necessitating the continual adaptation in plant defense strategies [128]. TALENs can be employed to edit genes associated with the plant immune system, thereby enhancing resistance to pathogens without compromising plant growth or productivity [12]. This targeted approach strengthens plant immunity while reducing dependence on chemical pesticides, contributing to more sustainable agricultural practices (Figure 3).

### 5.2. Transcription Activator-like Effector Nuclease-Mediated Modifications for Stress Tolerance

TALENs offer an effective tool for modifying the genetic makeup of plants to enhance their tolerance to various abiotic and biotic stresses. Abiotic stresses such as drought, salinity, and extreme temperatures are major challenges in modern agriculture. TALENs have been employed in targeting genes involved in water retention, osmotic regulation, and ion transport, all of which are essential for plant survival under stress conditions [129]. For instance, TALENs have been employed to modify genes involved in synthesizing osmoprotectants such as proline, which stabilizes cellular structures during drought conditions [130]. Similarly, genes encoding ion transporters, such as those for sodium and potassium, have been targeted to enhance salt tolerance in crops such as rice, barley, and tomato [131,132]. By enhancing the expression of these genes, TALENs can increase the ability of the plant to maintain cellular function under water-deficient or saline conditions, thereby increasing its resilience to drought and salinity stress.

Similarly, TALENs demonstrate effectiveness in addressing storage stress. Researchers employed a modified ligation-independent cloning method to efficiently insert TALEN monomer modules into plant vectors. These modular vectors were designed for genome editing, and following validation, TALEN pairs were used to generate stable transgenic rice lines via *Agrobacterium*-mediated transformation. A heterozygous mutant was obtained, and the mutation was successfully transmitted to the next generation. Molecular and protein analyses confirmed LOX3 deficiency and demonstrated enhanced seed storability. This work highlights the versatility of TALENs as a genome editing tool for enhancing agronomic traits and suggests that LOX3 has a limited effect on seed longevity [133].

Furthermore, TALENs have been used to modify genes involved in regulating plant responses to temperature stress. Heat stress causes protein denaturation and membrane destabilization, leading to cellular damage. To counteract this, plants produce HSPs that help to protect and repair damaged proteins. TALENs have also been successfully employed to target such genes in *Saccharomyces cerevisiae*. In this study, the TALEN-assisted multiplex editing (TAME) toolbox was utilized to enhance heat tolerance in *Saccharomyces cerevisiae* industrial strains. Two mutant strains exhibited a 1.2- to 1.3-fold increase in fermentation capacity under heat stress. Genome resequencing suggests that alterations in cell wall components, membrane proteins, and mitochondrial DNA contributed to the enhanced stress tolerance, despite the absence of direct modifications at the TALEN target sites. These findings highlight the potential of TAME for breeding stress-tolerant industrial strains and offer insights into the mechanisms underlying stress tolerance [98].

In addition to enhancing abiotic stress tolerance, TALENs are also being employed to enhance biotic stress resistance in plants. Plant pathogens—including bacteria, fungi, and viruses—can cause significant damage to crops, leading to yield losses and reduced quality. TALENs have been used to modify genes associated with plant immunity, thereby enhancing the ability of the plant to resist pathogen invasion. For example, TALENs have been employed to target mitogen-activated protein kinases, which play a central role in plant defense, with much of the foundational research conducted in *Arabidopsis thaliana*. In one study, the role of MPK3 in *Hordeum vulgare* (barley) was investigated in response to the bacterial elicitor flagellin peptide (flg22). TALENs were employed to generate MPK3 knock-out (HvMPK3 KO) barley lines, which exhibited reduced levels of defense proteins such as PR proteins and chitinases, along with diminished responsiveness to flg22. These plants also exhibited changes in root hair development and increased tolerance to flg22, highlighting the importance of MPK3 in the immune response of barley. Therefore, TALEN-mediated gene editing can enhance disease resistance in barley by modifying its immune system, ultimately leading to the development of healthier and more resilient crops [132].

Similarly, the successful use of TALENs for homology-directed (HR)-mediated gene editing in *Oryza sativa* has been reported, targeting the acetolactate synthase (OsALS) gene to introduce double point mutations conferring herbicide resistance. Following biolistic delivery of TALEN constructs and donor DNA into rice calli, nine HR events were obtained in the T_0_ generation, with varying OsALS genotypes and an editing efficiency of 1.4–6.3%. These HR-mediated gene edits were stably inherited in the T_1_ generation, with the edited plants exhibiting normal morphology and strong herbicide resistance. These findings validate TALEN-based genome editing as a viable strategy for developing pesticide-resistant rice and provide a foundation for future genome editing efforts using alternative nucleases [134].

Similarly, TALENs can be employed to target genes involved in producing antimicrobial peptides or regulating defense-related signaling pathways, such as those mediated by salicylic or jasmonic acids. In one investigation, TALENs were used to modify the HvMPK3 gene in *Hordeum vulgare* (barley), but the study does not specifically address the role of TALENs in upregulating jasmonic acid pathways. Instead, it demonstrates that knockout of the HvMPK3 gene affects the immune response of barley to the flg22, a well-known bacterial elicitor. The HvMPK3 knockout leads to differential expression of defense-related proteins, including chitinases and proteins from the thaumatin family, which are essential for plant defense. Proteomic analysis further shows changes in additional defense-related proteins, including some associated with jasmonic acid biosynthesis [132]. These advances in TALEN-mediated biotic stress tolerance have the potential to minimize the use of chemical pesticides, which pose risks to the environment and human health, thereby promoting more sustainable agricultural practices (Table 3).

## 6. Challenges and Limitations of TALENs

While TALENs have emerged as a powerful tool for enhancing secondary metabolite production in plants, their application is constrained by several challenges and limitations [12]. These include the inherent complexities of plant genomes, the high level of precision required for metabolic engineering, as well as the technical and regulatory barriers [140] that must be addressed to facilitate their broader adoption in agricultural and pharmaceutical settings. In particular, off-target effects, gene editing efficiency, and the risk of unintended consequences such as pleiotropic effects remain significant concerns that require careful management [141]. Furthermore, the regulatory frameworks governing genetically modified organisms (GMOs) and public acceptance of genetically engineered plants present further challenges to widespread implementation [142]. Despite these limitations, TALENs remain a promising genome editing tool, and ongoing research aims at enhancing their precision, minimizing off-target effects, and developing innovative strategies to address existing barriers.

### 6.1. Off-Target Effects

A major challenge in using TALENs for genome editing is the potential for off-target effects, where TALENs bind unintended DNA sequences and induce double-strand breaks at non-target loci [143]. Such off-target mutations can lead to undesirable genetic alterations, potentially causing unintended phenotypic changes in the edited plants [144]. While TALENs are generally regarded as more specific than those of other genome editing tools such as CRISPR-Cas9, the risk of off-target effects remains a concern, especially when editing complex genomes or employing multiple TALENs to target different loci. The occurrence of off-target mutations can disrupt genes unrelated to secondary metabolite production, potentially adversely affecting plant health, growth, or other essential metabolic processes [145]. To mitigate these risks, researchers have developed various strategies to enhance TALEN specificity, including using optimized TALEN design protocols, validating off-target effects through high-throughput sequencing techniques, and developing enhanced TALEN constructs with higher precision [146]. However, fully eliminating off-target effects remains a significant challenge that requires continuous refinement.

### 6.2. Regulatory and Ethical Concerns

The application of TALENs in plants—especially for modifying secondary metabolite production—also faces significant regulatory and ethical challenges. GMOs are governed by strict regulatory frameworks in many countries to ensure their safety for human health and the environment [147]. These regulatory frameworks often require comprehensive testing to assess potential risks, including allergenicity, toxicity, and ecological effects [148]. The approval process for GMOs can be lengthy and costly, potentially hindering the commercial application of TALENs in crop enhancement or pharmaceutical production [149]. Furthermore, public perception of genetically modified crops is often negative, especially in regions with strong opposition to GMOs owing to concerns about food safety, environmental risks, and corporate control of the food supply [150]. Ethical considerations surrounding the manipulation of plant genomes also significantly influence public opinion and regulatory frameworks. While TALENs offer precise control over gene editing, they raise critical concerns about the potential consequences of introducing genetically modified plants into the environment, especially those engineered for high-value secondary metabolite production [151]. Addressing these concerns will require transparent communication, comprehensive safety assessments, and active public engagement to foster trust in the technology.

### 6.3. Technical Barriers in High Throughput Screening

Another challenge in using TALENs to enhance secondary metabolite production lies in the technical difficulty of performing high-throughput screening across large populations of edited plants [8]. Efficient screening strategies are essential for identifying and selecting plants that exhibit the desired modifications in secondary metabolite production. However, generating stable TALEN-mediated edits in plants can be time-consuming and labor-intensive, especially in species with extended generation times or complex genomes [7]. While TALENs offer precise gene targeting, the overall efficiency of genome editing can vary depending on the plant species, the target gene, and the delivery method of the TALEN constructs. In many instances, achieving a high rate of successful gene edits with consistent outcomes remains a challenge. Developing more efficient delivery systems—such as nanoparticles or viral vectors—and the optimization of TALEN-mediated genome editing protocols remain ongoing areas of research aiming at enhancing overall success rates, but the ability to screen large populations of edited plants for secondary metabolite production remains a significant technical challenge. Addressing this challenge is essential for fully harnessing the potential of TALENs in crop enhancement and industrial-scale secondary metabolite production.

### 6.4. Editing Inefficiencies in Certain Crops or Genome Regions

**Figure 4:**
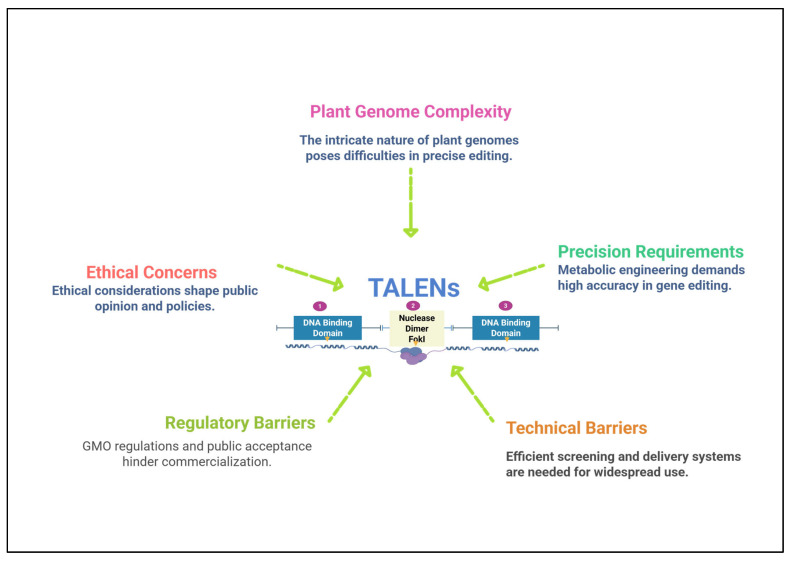
Challenges and limitations in TALENs (Transcription Activator-Like Effector Nucleases) based crop’s industrialization.

While TALENs are a powerful tool for genome editing, their efficiency can vary significantly across different plant species and even within specific regions of the genome [141]. Studies have shown that TALEN efficiency can be inconsistent across various crops, and certain genomic regions pose particular challenges [152]. For example, in crops such as maize and rice, TALENs have demonstrated lower editing efficiency compared to other genome-editing technologies like CRISPR/Cas9 [153]. This variability is influenced by several factors, including the complexity of the plant genome, the target gene’s location, and the presence of repetitive DNA sequences, which can impede precise targeting by TALENs [29]. In particular, certain regions of the genome, such as those containing high GC content or gene-rich areas, are more difficult to edit with TALENs [154] (Figure 4).

## 7. Future Directions and Prospects

Despite the development of prime editing and base editing technologies, TALENs remain an important tool in genome editing, particularly in plant systems. These newer technologies, while offering significant precision in modifying individual nucleotides, do not yet possess the same flexibility for making large-scale modifications [155]. TALENs can introduce complex genetic changes, such as deletions, insertions, and multi-locus modifications, which are often necessary for optimizing metabolic pathways and enhancing secondary metabolite production in plants [156]. This capacity to target multiple loci simultaneously is particularly beneficial for traits governed by polygenic pathways, making TALENs a viable option for applications requiring comprehensive genetic alterations [157].

Applying TALENs in enhancing plant secondary metabolite production shows significant promise, but numerous opportunities remain for further development and refinement. As the field of plant metabolic engineering progresses, TALENs technology holds significant potential to revolutionize the production of high-value secondary metabolites in agricultural and pharmaceutical industries [10]. With increasing demand for high-value plant-derived compounds especially those with therapeutic potential TALENs offer a precise and effective approach for enhancing metabolic pathways to enable economically viable production of these bioactive molecules [158]. Ongoing advancements in TALEN technology such as enhanced specificity, editing efficiency, and delivery systems are expected to expand their applications across various areas of plant biotechnology.

### 7.1. Next-Generation TALENs: Improved Specificity and Efficiency

Developing next-generation TALENs aims to address some limitations associated with the current version of the technology, especially challenges related to target specificity and editing efficiency [159]. While TALENs are generally more specific than those of other genome-editing tools such as CRISPR-Cas9, off-target effects remain a significant concern. To enhance the precision of TALENs, researchers are refining the DNA-binding domain to increase target affinity and specificity, thereby minimizing the possibility of unintended binding and off-target mutations [160]. Recent advancements in TALEN design include the use of extended repeat regions within the DNA-binding domain and structural optimizations that enhance sequence-specific recognition. In addition to enhancing specificity, researchers are also striving to increase the editing efficiency of TALENs [161]. Strategies such as enhancing FokI nuclease dimerization and optimizing delivery methods aim to achieve higher rates of successful genome modifications, an essential step toward scaling up TALEN-based applications in plant metabolic engineering [162].

### 7.2. Integration with Other Genetic Engineering Tools

While TALENs are effective as standalone tools, their full potential can be harnessed when integrated with other genome-editing technologies, such as CRISPR/Cas9, ZFNs, or RNA interference (RNAi) [29,163]. Each of these technologies has its strengths and limitations, and their combined application enables researchers to leverage the unique advantages of each tool in enhancing the precision, versatility, and efficiency of genetic modifications. For example, CRISPR/Cas9 is widely recognized for its simplicity and high efficiency in targeting specific genomic sequences [164]. When combined with TALENs, CRISPR/Cas9 can facilitate the simultaneous targeting of additional loci or enable multiplexed edits within a single transformation event, thereby enabling the modification of multiple genes involved in secondary metabolite pathways [165]. Alternatively, RNAi can be employed to modulate gene expression in a more reversible and tunable manner, complementing the more permanent genetic modifications introduced by TALENs [166]. Integrating TALENs with tools such as RNAi enables the development of more sophisticated and robust strategies for enhancing secondary metabolite production, allowing for precise modulation of plant metabolic pathways, leading to optimized profiles of bioactive compounds [44]. This integrated approach holds significant promise for enhancing the efficiency of secondary metabolite production, especially for compounds with complex biosynthetic pathways.

### 7.3. Integration with Omics Data, Machine Learning and AI-Assisted Genome Design

The Integration of TALENs with multi-omics data (such as genomics, transcriptomics, proteomics, and metabolomics) can augment the accuracy and scope of synthetic biology applications. For instance, combining TALENs with genomic and epigenomic data allows researchers to examine how targeted genome editing affects gene regulation and chromatin structure. This approach could be used to understand the effects of gene editing on epigenetic modifications such as DNA methylation and histone modifications, which influence gene expression and cellular function [167].

Combining TALENs with transcriptomics and proteomics provides valuable insights into impact of genetic modifications on gene expression and protein production. For instance, an application of TALENs in combination with RNA-Seq and proteomics allows the details analysis of how specific genetic modifications effect various cellular pathways and metabolic processes. Attempt have been made to use AI-assisted genomic prediction models to improve breeding programs, potentially incorporating TALENs in plant and crop genome editing to optimize traits such as disease resistance and yield [168].

Similarly, the use of AI-assisted genome editing has been pragmatic in the field of plant breeding. AI algorithms can facilitate the design of TALEN constructs to accomplish specific traits, such as increased crop resilience or improved nutritional value. Investigators explored how artificial intelligence can revolutionize plant breeding by integrating AI with genome-editing tools like TALENs, providing more efficient and precise modifications [169].

### 7.4. Industrial Applications of Enhanced Secondary Metabolites

The successful application of TALENs with other genome editing technologies, including ZFN, TALEN, and CRISPR/Cas, can facilitate enhanced secondary metabolite production in plants, opening promising avenues for industrial and commercial application [11]. Currently, many high-value secondary metabolites—such as alkaloids, terpenoids, and flavonoids—are extracted from plants in limited quantities or synthesized chemically, which can be expensive and environmentally damaging. TALEN-mediated enhancements could significantly enhance the yield and quality of these compounds, offering a more sustainable and cost-effective production approach [170,171]. For example, the ability to increase the production of vincristine and vinblastine in *Catharanthus roseus* through TALENs could enable a more reliable and affordable supply of these essential anticancer drugs, which remain in high demand but are currently produced in limited quantities. Similarly, terpenoids—used in fragrance, flavoring, and biofuel production—could be synthesized in higher concentrations, making plant-based production more competitive with synthetic approaches. Beyond pharmaceuticals, TALENs could also be employed to enhance the production of essential oils, antioxidants, and other nutraceuticals that are increasingly valued for their health-promoting properties [172,173]. Developing genetically modified plants with enhanced profiles of bioactive compounds could have a transformative impact across various sectors, including medicine, agriculture, and biotechnology.

## 8. Conclusions

TALEN technology demonstrates significant potential for enhancing the production of secondary metabolites in plants by offering a precise, targeted approach for modifying key genes involved in biosynthetic pathways. As outlined in this review, TALENs have been successfully applied across various plant species—especially medicinal plants—to boost the synthesis of high-value bioactive compounds such as alkaloids, terpenoids, flavonoids, and phenolic acids. These compounds have broad applications in pharmaceuticals, agriculture, and other industries, and their enhanced production through TALEN-mediated genome editing offers a promising strategy to improve the yield and quality of these metabolites. Looking ahead, integrating TALENs into plant metabolic engineering holds significant potential for advancing sustainable agriculture and developing high-value plant-derived pharmaceuticals. By enabling the large-scale production of high-quality, plant-derived compounds, TALENs offer a solution to the growing global demands for natural products while reducing dependence on chemical synthesis and large-scale cultivation. As research in TALENs technology continues to advance, future research efforts will probably focus on optimizing the efficiency, target specificity, and scalability of these genetic modifications for commercial applications. The continued refinement of TALEN-based approaches could revolutionize the production and utilization of plant secondary metabolites, fostering more sustainable and efficient agricultural practices as well as pharmaceutical manufacturing systems.

## Figures and Tables

**Table 1:** Key features and comparisons of TALENs, CRISPR-Cas9, and ZFNs in plant genome editing.

Feature	TALENs	CRISPR-Cas9	ZFNs	Citations
Specificity	High specificity due to protein-DNA interactions, reducing off-target effects	Can have off-target effects due to reliance on RNA sequences (guide RNAs)	Similar to TALENs in specificity but less precise in some contexts	[36,45]
Target Range	Can target a broader range of DNA sequences without sequence motif restrictions	Limited by PAM (Protospacer Adjacent Motif) sequence availability	Limited by target DNA sequence and zinc-finger binding specificity	[45]
Suitability for Complex Genomes	Ideal for editing complex genomes where off-target mutations are a concern	Less suitable for complex genomes due to potential off-target effects	Can be used for complex genomes, but less flexible than TALENs	[41,46]
Risk of Genomic Rearrangements	Lower risk of genomic rearrangements or insertions at off-target sites	Higher risk of off-target genomic rearrangements	Higher risk of off-target genomic rearrangements	[47,48]
Flexibility in Genome Editing	High flexibility, can target nearly any sequence in the genome	Limited by PAM sequences, reducing flexibility in certain regions	Can target a variety of sequences, but less flexible than TALENs	[10,12]
Cost and Construction	Expensive and time-consuming to design and construct	Easier and more cost-effective to design and implement	Expensive and time-consuming to design and construct	[36,37]
Ease of Use	Labor-intensive, requires iterative design and validation	Simpler design, widely accessible and easier to use	Requires extensive optimization, less user-friendly than CRISPR-Cas9	[37,49]
Application in Plant Species	Effective in both dicots and monocots, even with complex or poorly characterized genomes	Variable success in different plant species due to PAM sequence availability	Effective, but less flexible than TALENs for targeting specific plant genomes	[41]

**Table 2:** Applications of TALENs in modifying secondary metabolite pathways in plants.

Strategy/Approach	Target Genes/Enzymes	Examples of Secondary Metabolites	Impact of TALEN Modification	Reference
Enhancing Production by Targeting Key Enzymes	Terpene Synthases (e.g., TPS1, TPS2), Flavonoid Synthases (e.g., CHS, F3H), Cytochrome P450s (e.g., CYPs)	Terpenoids (e.g., limonene, artemisinin), Flavonoids, Phenylpropanoids	TALENs can directly target key genes in the biosynthesis of terpenoids and flavonoids, enhancing the production of valuable secondary metabolites for medicinal, fragrance, and flavor uses	[45]
Manipulating Precursor Pathways	Amino Acid Synthesis Genes (e.g., TRP1, TAT1), DAHPS (Shikimate Pathway), AroB (Aromatic Pathway)	Alkaloids (e.g., nicotine, morphine), Amino Acid-Derived Compounds (e.g., tryptophan derivatives)	TALENs can modify precursor pathways, enhancing the availability of amino acids and redirecting metabolic flow to increase alkaloid and other valuable secondary metabolite production	[82]
Targeting Regulatory Proteins	MYB Transcription Factors (e.g., MYB46, MYB75), bHLH Factors, WRKY Transcription Factors	Various secondary metabolites (e.g., terpenoids, flavonoids, alkaloids)	TALENs can be used to activate or suppress transcription factors involved in regulating secondary metabolic pathways, enabling increased production of targeted metabolites	[83]
Multiplex Editing for Enhanced Production	Multiple Genes in the Flavonoid Pathway (e.g., CHS, F3H, F3′H, DFR, ANS)	Flavonoids, Lignans, Alkaloids	TALENs can be used to target multiple genes within the same pathway, leading to an enhanced overall yield of secondary metabolites while improving plant traits	[84,85]
Combination with Metabolic Engineering	Shikimate Pathway Enzymes (e.g., DAHPS, AroB), Precursor Enzymes (e.g., Tyrosine Decarboxylase, Tryptophan Synthase)	Aromatic compounds (e.g., flavonoids, lignans), Alkaloids	TALENs can be combined with metabolic engineering to optimize precursor production, enhancing the efficiency of secondary metabolite biosynthesis	

**Table 3:** TALEN-mediated modifications for enhancing plant stress tolerance.

Stress Type	Targeted Mechanism/Genes	Example of TALEN-Mediated Modification	Impact of Modification	References
Abiotic Stress	Water Retention/Osmotic Regulation Genes encoding osmoprotectants (e.g., Proline Synthesis Genes, P5CS gene)	TALENs used to modify genes involved in proline biosynthesis in rice and tomato to enhance drought tolerance	Increased proline production	[135]
Abiotic Stress	Ion Transport Genes encoding Sodium-Potassium Transporters (e.g., HKT1 for sodium uptake, NHX1 for vacuolar Na+/H+ exchange)	TALENs targeting genes for improved salt tolerance in rice and tomato, specifically targeting HKT1 and NHX1 genes	Enhanced salt tolerance	[136]
Abiotic Stress	Temperature Stress (Heat and Cold Tolerance) Genes encoding Heat Shock Proteins (HSPs) and Antifreeze Proteins (e.g., HSP70, COR15a)	TALENs used to modify HSP70 and COR15a genes in tobacco and Arabidopsis to enhance heat and cold tolerance	Increased expression of heat shock proteins (HSPs)	
Biotic Stress	Plant Immunity Genes, Genes encoding Antimicrobial Peptides (AMPs), Defensin-like Proteins, Defense Signaling Pathways (e.g., SA and JA pathways)	TALENs used to enhance resistance to Fusarium wilt and bacterial blight in tomato by targeting defense-related genes like PR1 (pathogenesis-related protein) and PR2 (glucanase)	TALEN-induced overexpression of defense-related genes like PR1 and PR2	[137,138]
Biotic Stress	Pathogen Recognition Genes involved in Pattern Recognition Receptors (PRRs) (e.g., FLS2, EFR) for pathogen detection.	TALENs employed to enhance recognition of PAMPs (Pathogen-Associated Molecular Patterns) in Arabidopsis and rice by targeting FLS2 (flagellin receptor) and EFR (elongation factor receptor) genes.	Enhanced pathogen recognition	[139]

## Data Availability

No new data were created or analyzed in this study.
